# The Dynamics of the Skin’s Immune System

**DOI:** 10.3390/ijms20081811

**Published:** 2019-04-12

**Authors:** Alan V. Nguyen, Athena M. Soulika

**Affiliations:** 1 Institute for Pediatric Regenerative Medicine, Shriners Hospitals for Children Northern California, Sacramento, CA 95817, USA; alvnguyen@ucdavis.edu; 2 Department of Dermatology, School of Medicine, University of California Davis, Sacramento, CA 95817, USA

**Keywords:** skin resident immune cells, skin immune responses, inflammation, wound healing, impaired wound healing, scarring

## Abstract

The skin is a complex organ that has devised numerous strategies, such as physical, chemical, and microbiological barriers, to protect the host from external insults. In addition, the skin contains an intricate network of immune cells resident to the tissue, crucial for host defense as well as tissue homeostasis. In the event of an insult, the skin-resident immune cells are crucial not only for prevention of infection but also for tissue reconstruction. Deregulation of immune responses often leads to impaired healing and poor tissue restoration and function. In this review, we will discuss the defensive components of the skin and focus on the function of skin-resident immune cells in homeostasis and their role in wound healing.

## 1. Introduction 

The skin is a complex organ, crucial for maintaining important functions for host physiology such as preventing fluid loss, stabilizing body temperature and relaying sensory inputs [1,2]. In addition, it harbors a highly specialized immunological niche crucial for the maintenance of tissue homeostasis, defense, and repair. In this review, we will discuss the structure of the skin and its intrinsic defensive mechanisms, with a focus on the skin-resident immune cells and their contributions to tissue repair.

## 2. Skin Structure 

The skin is structured into three layers: the epidermis, the dermis, and subcutaneous fat tissue. The epidermis, the outermost layer of the skin, is subdivided into the stratum corneum, stratum lucidum, stratum granulosum, and stratum basale. The stratum corneum contains corneocytes, which are terminally differentiated keratinocytes. These cells are continuously replenished by keratinocytes localized in the stratum basale [1]. The stratum lucidum is a thin, clear layer of dead keratinocytes. Instead of keratin, keratinocytes in the stratum lucidum contain eleidin, a clear intracellular protein, which gives this layer its transparent appearance. The stratum granulosum is a thin layer between the stratum lucidum and stratum basale. Keratinocytes in the stratum granulosum contain cysteine- and histidine-rich granules, which bind keratin filaments together [2]. The stratum basale contains basal keratinocytes, immune cells such as Langerhans cells and T cells, and melanocytes that provide the skin with pigmentation. 

Beneath the epidermis is the dermis, which is further categorized into papillary and reticular sub-layers. In humans, the papillary dermis forms extensions that reach out to the epidermis and contain capillaries that facilitate the transport of nutrients [3]. Reticular dermis contains skin appendages such as hair follicles, sebaceous glands, and sweat glands. The reticular dermis is significantly thicker than the papillary dermis due to the dense concentration of collagenous and reticular fibers that are interwoven within this layer. Both dermal layers house fibroblasts, myofibroblasts, and immune cells such as macrophages, lymphocytes, and mast cells. Fibroblasts synthesize an extracellular matrix consisting of collagen, proteoglycans, and elastic fibers that provide the structural integrity of the dermis [4]. 

Underlying the dermis is the subcutaneous fat. This layer consists of fibrocytes and adipocytes and is rich in proteoglycans and glycosaminoglycans, which confer mucus-like properties to the layer [5]. Skin adipose tissue stores energy in the form of fatty acids and functions as an endocrine organ important for glucose homeostasis and lipid metabolism [6,7,8]. This layer also produces a variety of mediators such as growth factors, adipokines, and cytokines, and contains multiple immune cells [9]. In addition, the subcutaneous fat serves as an insulating layer for the body, as fat is a poor conductor of heat.

Murine models are commonly used in dermatological research. These models are of great value and mimic many pathological aspects observed in human skin disorders. However, it is important to note that there are differences in the skin structure between mice and human. The thickness of murine epidermis is thinner than that of humans’, which depending on the area of the body, is between six and 32 layers [10]. The epithelial architecture of human skin contains rete ridges, which are extensions that project into the underlying tissue, and do not exist in murine skin [11]. In contrast to humans, the murine dermis lack sweat glands, excluding the mammary glands in female mice. Furthermore, mice contain a dermal muscular layer, also known as the panniculus carnosus, which is absent in humans [10]. The skin of other animals such as pigs more closely resembles that of humans [12], so these animals are increasingly employed in experimental settings. However, murine models are still widely used due to easy access and lower costs and have proved effective for the study of inflammatory diseases of the skin.

## 3. The Skin as an Immune Organ

One of the main functions of the skin is to protect the host from invasion, and it does so by employing physical barriers, biomolecules, and an intricate network of resident immune and non-immune cells and skin structures. Furthermore, in the absence of a challenge, resident immune cells promote skin physiological functions. Below we will describe the strategies used by the skin to defend and protect the host, and their contributions to tissue homeostasis. 

### 3.1. Physical Barriers

The barrier function of the epidermis is mainly mediated by corneocytes in the stratum corneum. These cells are organized in a “bricks and mortar” manner, interspersed by lipids such as ceramides, cholesterol, and free fatty acids [13]. Each corneocyte contains a lipid envelope linked to keratin filament bundles that fill the intracellular compartments of the corneocyte, thus strengthening its rigidity [14]. The stratum corneum is composed of three layers and it is both an outside‒in barrier to prevent the entry of foreign substances and microorganisms, and an inside‒out barrier to prevent water loss [15]. 

The formation of the physical barrier of the skin function depends on junction adhesion molecules and tight junction proteins. Disruptions in the expression or function of these components will result in improper barrier formation or skin disorders. Junctional adhesion molecules (JAMs), claudins, zonula occludins-1 (ZO-1), and occludins are found in epidermal layers. Claudin-1, claudin-7, and JAM-A are found in all layers of the human epidermis [16], while claudin-1, claudin-12, and JAM-A are ubiquitous in murine epidermal layers [17,18]. The importance of tight junction proteins in skin function was demonstrated by Furuse et al., who showed that deletion of claudin-1 in mice led to death shortly after birth as a result of cutaneous defects ultimately leading to significant loss of fluids [19].

Impairment of the components of the skin’s physical barriers can contribute to inflammatory conditions in the skin. For example, the skin of patients with atopic dermatitis exhibits reduced expression levels of ZO-1 and claudin-1 [20], and knockdown of claudin-1 in mice induces a psoriasis-like condition [21].

### 3.2. Biomolecules of the Skin 

Antimicrobial peptides (AMPs) and lipids are the main classes of biomolecules that participate in skin defense by disrupting bacterial membranes [22,23,24]. 

AMPs are amphipathic peptides and are expressed constitutively or induced after cell activation in response to inflammatory or homeostatic stimulation. The most thoroughly studied AMP families in human skin are the defensins and the cathelicidins, which are produced by a variety of cells in the skin such as keratinocytes, fibroblasts, dendritic cells, monocytes, and macrophages, and sweat and sebaceous glands [22,23]. AMPs are produced as propeptides and become active after proteolytic cleavage. In contrast to defensin family members, which are produced by distinct genes, there is only one gene associated with cathelicidins (cathelicidin antimicrobial peptide—CAMP) [25]. A number of active peptides can be generated via proteolytic cleavage of the inactive CAMP product, but the most studied cathelicidin is LL-37 [26]. AMPs may act synergistically and have broad activity against microbial species [27,28]. A recent study suggests certain AMPs activity may be pathogen-specific [28]. Recently, due to increased resistance to antibiotics, a focus of active research has been the evaluation of AMPs as therapeutics against infections [22].

Interestingly, AMPs have roles in modulating host immune responses. Human LL-37 was shown to induce differentiation of monocyte-derived dendritic cells, subsequent cytokine production, and expression of the co-stimulatory molecule CD86 [29]. LL-37 and β-defensins can also serve as alarmins for keratinocytes by inducing their proliferation and migration [30]. Furthermore, human LL-37 exerts its alarmin effects on immune cells in a synergistic manner with other inflammatory mediators, such as IL-1β [31]. Human α- and β-defensins serve as chemoattractants for activated neutrophils, memory and naïve T cells and immature dendritic cells [32], while β-defensins 3 and 4 can recruit monocytes and macrophages [33]. The murine version of β-defensin 2 induces dendritic cell maturation by upregulating the expression of co-stimulatory molecules and the antigen presentation molecule MHCII [34]. 

In addition, AMP functions have been associated with the processes of aging and memory [26,32,35]. Interestingly, LL-37 has been shown to exert proangiogenic effects and may also play a role in tissue repair [36].

Lipids such as sphingomyelin, glucosylceramides, and phospholipids are intermediate molecules, readily converted into sphingosine and dihydrosphingosine, which exert antimicrobial activity against certain bacterial strains such as *Staphylococcus aureus*, *Streptococcus pyogenes*, *Micrococcus leutus*, and *Proprionibacterium acnes* [37]. These lipids are stored in lamellar bodies found in corneocytes in the stratum corneum [38].

Sebocytes residing in the sebaceous glands produce sebum, which is rich in lipids such as triacylglycerol, wax esters, non-esterized fatty acids, and squalene. While the functions of sebum are not fully understood, there is a consensus that sebum serves as a “seal” for the hair follicles, thus preventing entry of microbes into the deeper layers of the skin. Intriguingly, a previous study demonstrated that the AMP dermcidin is expressed by sebocytes, suggesting that sebum exerts defensive functions [39]. Furthermore, sebum can be further processed into free fatty acids by skin commensal bacteria [40,41], and in humans, sebum-derived free fatty acids induce β-defensin 2 expression by sebocytes, further suggesting that sebaceous glands serve an innate defensive role [42].

### 3.3. pH of the Skin 

The pH of human skin is 5.4‒5.9, which makes the skin an inhospitable environment for potential pathogens [43,44]. Furthermore, the dramatic difference in pH levels between the skin and the blood (pH = 7.4) serves as a secondary defensive mechanism in the event that microbes breach the skin tissue and enter the circulation. There are various ways that the skin maintains a low pH.

Filaggrin, a filament-associated protein that binds keratin fibers, is broken down into histidine, which is further processed by histidase, expressed by corneocytes into the acidic metabolite trans-urocanic acid [45]; this has been implicated in the acidification of the stratum corneum [46]. Fatty acids produced in the stratum corneum also alter the acidity of the skin [41,44]. In addition, sweat glands produce acidic electrolytes and lactic acid, which lowers the pH of the skin [47] and promotes epidermal turnover [48].

Furthermore, the physiological pH of the skin is hospitable for commensal bacteria such as *Staphylococcus epidermidis*, preventing pathogenic strains such as *Staphylococcus aureus* from establishing infections in the host [49,50]. 

### 3.4. Immune Cells of the Skin 

Skin-resident immune cells promote tissue function in homeostasis and act as sentinels by actively sampling environmental antigens. Both myeloid and lymphoid cell subsets are found in the skin in steady state (Table 1). Some of these resident immune cells migrate to lymph nodes to either induce peripheral tolerance to tissue self-antigens or initiate robust immune responses. In the event of a challenge, such as infections or tissue injury, immune cells resident in the skin and those infiltrating from the periphery interact to create an intricate defense network to resolve the insult and restore the tissue to its original state. In this section we will describe the functions of myeloid and lymphoid cell subsets that are resident to the skin.

#### 3.4.1. Myeloid Cells 

Skin-resident myeloid cells include Langerhans cells, dermal dendritic cells, macrophages, mast cells, and eosinophils. Neutrophils are rarely found in healthy skin and thus are not “skin-resident cells.” However, neutrophils populate the skin in inflammatory conditions and after a wound, and they will be discussed in the wound healing sections of this review. 

Skin-resident myeloid cells contribute to skin homeostasis by secreting growth factors needed for the survival of keratinocytes, fibroblasts, and endothelial cells. In addition, they maintain optimal tissue function by phagocytosing debris and apoptotic cells, supporting vasculature integrity, and promoting tolerance. 

In inflammatory conditions, myeloid cells respond immediately and produce pro-inflammatory mediators that drive the activation of cells in the local vicinity and infiltration of the affected site by peripheral immune cells. Skin myeloid cells also serve as a liaison between the innate and adaptive immune system. 

##### Langerhans Cells 

Langerhans cells (LCs) are the sole myeloid cell type in the epidermis. Phenotypically, LCs are characterized by high expression of MHCII and the presence of langerin+ Birbeck granules [51,82]. LC maintenance depends on keratinocyte-derived IL-34 [82,83,84,85], one of the ligands for the colony stimulating factor-1 receptor (CSF-1R). CSF-1R is constitutively expressed on LCs and global deletion of this receptor results in a total lack of LCs [85,86,87]. CSF1 is dispensable for LC maintenance as CSF1 knockout mice retain their LC populations [87]. LCs are absent in mice deficient in inhibitor of DNA binding 2 (Id2) [88], or Runt-related transcription factor 3 (Runx3) [89]. Both Id2 and Runx3 are involved in TGFβ signaling [88,89], and TGFβ deficiency also results in the complete loss of LCs. 

LCs are derived from two sources, the extra-embryonic yolk sac and fetal liver monocytes; so far, there are no functional attributes unique to each niche [90]. LCs renew from a local progenitor cell [91], as shown for the first time by Merad et al., via bone marrow chimeric mice in which donor CD45.2+ bone marrow cells were injected into lethally irradiated CD45.1+ recipient mice. After reconstitution and BrdU administration, LCs in the recipient mice were of host origin (CD45.1+), and incorporated BrdU, indicating that LCs are self-replenishing during homeostasis [91,92]. In inflammatory conditions, bone-marrow-derived cells are also able to give rise to short-lived or long-lived LCs in an IL-34-independent manner [85,93]. Long-lived bone-marrow-derived LCs are Id2-dependent [87,94]. 

In homeostasis, LCs anchor themselves within the epidermis through interactions between epithelial cell adhesion molecules (EpCAM) or E-cadherin expressed on LCs and E-cadherin expressed by keratinocytes [51,95,96]. Furthermore, autocrine and paracrine TGFβ signaling restricts LCs in the epidermis by regulating the expression of E-cadherin on LCs [97,98], and increases phagocytic behavior in LCs during steady state [99]. While anchored, LCs sample antigens and, upon activation, they can extend their processes from the cell body outward to the stratum corneum or below toward the stratum basale [51]. LCs participate in tight junction formation and thus can sample the microenvironment without damaging the barrier [51].

LCs are migratory cells and continually travel to the skin draining lymph nodes to promote tolerance in homeostasis [93] or to initiate adaptive immune responses [100,101,102]. To migrate from the epidermis to local lymphatic vessels, LCs disengage the adhesion interaction between E-cadherin and EpCAM/E-cadherin. The release of E-cadherin is mediated by β-catenin, which is associated with the intracellular tail of E-cadherin and provides a link between the adhesion molecule and the cytoskeleton [103]. LCs express matrix metalloprotease 9 (MMP9), which allows for the restructuring of the dermal collagen network to create a path for LC migration toward the lymphatic vessel [88]. Inhibition of MMP9 activity reduces LC migration from murine and human epidermal sheets [104]. The migration of LCs (and also of dermal dendritic cells) through the dermis is mediated via CXCR4 signaling after binding to its cognate chemokine CXCL12, produced by dermal fibroblasts [105].

In the presence of inflammatory mediators and other activators such as pathogen-associated molecular patterns (PAMPs) and danger-associated molecular patterns (DAMPs), LCs upregulate co-stimulatory molecules and migrate from the epidermis en masse to the draining lymph nodes, where they prime adaptive immune responses in a manner equivalent to that of conventional dendritic cells [51,106,107,108,109]. TGFβ signaling is disrupted during inflammation, thus also promoting LC migration from the epidermis [97]. LCs are professional antigen-presenting cells and activate both CD8+ cytotoxic T lymphocytes and CD4+ helper T lymphocytes. In humans, efficient antigen presentation by LCs is dependent upon caveolin-1, a scaffold protein that serves a multitude of functions including transport of lipids, signal transduction, and membrane trafficking [110,111]. LCs can also exert immunoregulatory and tolerogenic functions [51,112,113]. They were shown to promote tolerance to keratinocyte antigens in the skin by promoting the expansion of desmoglein 3-specific T regulatory cells [74], while LC depletion led to the formation of anti-desmoglein 3 antibodies resulting in dermatitis in mice [75]. 

However, LCs are also known to play roles in various skin diseases such as psoriasis, atopic dermatitis, and non-melanoma skin tumors [114,115,116].

##### Dermal Dendritic Cells 

Dendritic cells that reside in the dermis are known as dermal dendritic cells (dDCs). In a similar manner to LCs, dDCs migrate to the lymph nodes, and are professional antigen-presenting cells efficient at priming adaptive immune responses [117,118]. It was recently shown that dDCs can induce tolerance to topically applied antigens encountered in the hair follicles [119].

Unlike LCs, all dDCs are derived from progenitors originating from the bone marrow, with replenishment occurring roughly every seven days [120,121]. The two main subsets of dDCs are the conventional dendritic cell type 1 (cDC1) and the conventional dendritic cell type 2 (cDC2) [122,123]. Murine cDC1s express langerin (CD207), low levels of the fractalkine receptor CX3CR1, the chemokine receptor XCR1, and CD103 (ITGAE), but do not express the myeloid marker CD11b. cDC2s are the most abundant subtype of dDCs in murine skin and express CD11b, CD301b, also known as macrophage galactose-type C-type lectin 2 (MGL2), low levels of langerin [121], and higher levels of CX3CR1 in comparison to cDC1s [124]. Although a CD103+CD11b+ DC population exists in the intestines [125,126], expression of CD11b and CD103 is mutually exclusive in skin dendritic cells. In the mouse, both cDC1s and cDC2s require stimulation via Flt3 ligand (Flt3l) and CSF2 [127] for development. For population maintenance, cDC1s require basic leucine zipper transcriptional factor 3 (Batf3) and IRF8 [128,129], while cDC2s require IRF4 [129,130,131]. A subset of dendritic cells expressing CD8α exists in lymphoid tissues but not in the skin [123,132]. These cells are capable of cross-presentation, a process during which extracellular antigens, normally processed via the MHCII pathway, are also presented via the MHCI pathway. Like CD8+ DCs, murine dermal cDC1s can cross-present antigens [133,134]. 

In the mouse, cDC1s are associated with Th1 responses [135,136], while cDC2s are associated with Th2 and Th17 [125,137,138,139]. Th1 responses are mainly specific for intracellular pathogens, while Th2 and Th17 are for extracellular pathogens. Furthermore, both Th1 and Th17 cells are associated with autoimmune disorders, while Th2 cells are associated with asthma and allergic disorders. Mice deficient in *Irf8*, in which cDC1s are depleted, generate reduced numbers of Th1 cells in the skin in response to cutaneous infection with herpes simplex virus (HSV) [135]. Kruppel-like factor 4 (Klf4) has been shown to be required for maintaining conventional DC populations in the spleen [140]. Intriguingly, Tussiwand et al. demonstrated that expression of Klf4 in dDCs is critical for the induction of Th2 responses in vivo [141].

dDCs are implicated in maintaining homeostatic interactions between the host and skin-resident commensal bacteria. Naik et al. showed that mice deficient in Batf3 (necessary for cDC1) exhibit reduced populations of CD8+ IL-17+ T cells specific for *Staphylococcus epidermidis*, suggesting that cDC1s control the generation of commensal-specific T cells in the skin [52]. 

Plasmacytoid DCs (pDCs) are a DC subset, found in the skin exclusively under inflammatory conditions. pDCs are mass producers of IFNα, which is essential for viral defense [123]. In mice, pDCs can be identified by the marker B220, which is also expressed by B lymphocytes [142,143,144]. In addition to their antiviral functions, pDCs have been implicated in autoimmune skin disorders such as psoriasis [145]. Interestingly, a recent study showed that depletion of pDCs using CLEC4C-DTR mice treated with bleomycin to induce skin fibrosis exhibited a decrease in dermal thickness and collagen content when compared to WT control mice [142], suggesting that pDCs play a role in fibrosis.

Human dDCs are subdivided into cDC1 (CD141+), cDC2 (CD1c+), and CD14+ dDCs [123]. cDC1s co-express CD304 (neuropilin-1), XCR1, and CD370 (CLEC9A). Unlike murine dDCs, human dDCs do not express langerin [123,146]. Human cDC1s can cross-present in a similar manner to murine cDC1s [147] and are potent in inducing Th1 responses [148]. cDC2s and CD14+ dDCs co-express CD11b and CX3CR1. Interestingly, human cDC1s and cDC2s are both capable in inducing Th2 responses [148]. Human pDCs express CD304, CD303 and CD123 and like their murine counterparts are only found in inflamed skin. Remarkably, a recent study showed that human peripheral blood pDCs upregulate the expression of B cell maturation antigens upon activation via TLR9 signaling, suggesting that pDCs play a role in the maintenance of plasma cells, in addition to their inflammatory functions [149]. 

##### Macrophages 

Macrophages are found in the dermal layer of the skin and require IL-34 for survival [86,150]. Two sources of dermal macrophages have been identified so far. The first source is embryo-derived progenitors that seed the skin prenatally and are self-renewing in a similar fashion to LCs [126]. The second and major source of dermal macrophages is circulating monocytes (monocyte-derived macrophages) that mature once they reach the skin. This population replenishes roughly every 10 days [151,152]. Monocytes that give rise to dermal macrophages express lymphocyte antigen 6C (Ly6C), and home to the skin in a CCR2-dependent manner [129]. As monocytes mature into skin-resident macrophages, the expression of CCR2 is downregulated [153]. CD64 expression is prominent on dermal macrophages and is used as a marker to differentiate them from the dDCs [152,153]. CD36, DC-SIGN, and IL-10 are highly expressed by macrophages isolated from healthy skin, suggesting that they adapt an immunoregulatory phenotype [153,154]. In steady state, macrophages remove cellular debris [129,153], and have also been implicated in homeostatic hair regeneration [54,155]. Macrophages can be localized at post-capillary venules in the skin and secrete chemokines that drive the recruitment of neutrophils [156]. However, the depletion of macrophages does not translate into a reduction of neutrophils in the skin in wounds [152,157], indicating that dermal macrophages are dispensable for neutrophil infiltration. 

Macrophages are plastic, and one way to categorize their effector functions is as pro-inflammatory “M1” or anti-inflammatory/pro-repair “M2.” It is important to note that bona fide M1 and M2 macrophages are generated only in vitro, and in vivo macrophages may simultaneously express M1 and M2 markers. Although investigators are beginning to shift away from the M1/M2 macrophage paradigm [158], we will use this nomenclature to describe and categorize the functions of macrophages as pro-inflammatory or anti-inflammatory/pro-repair. M1 macrophages express inducible nitric oxide synthase (iNOS), and secrete inflammatory cytokines such as TNFα, IL-1β, and IL-6 [159,160]. M2, or “alternatively activated” macrophages, adopt an anti-inflammatory and/or pro-repair phenotype. M2 macrophages can be further subdivided into M2a, M2b, M2c, and M2d. M2a are known to be pro-fibrotic due to TGFβ production [161]. M2b express the co-stimulatory molecule CD86 and as such they are the most pro-inflammatory of the M2 subsets. M2c macrophages are induced by IL-10 or TGFβ, express the Mer tyrosine kinase (MerTK) and promote neovascularization [162], and have high scavenging and debris clearing activity. M2d cells are responsive to IL-6 and exhibit a few of the properties of tumor-associated macrophages such as secretion of IL-12 and IL-10 [162,163,164,165]. M2d cells are also known to express high levels of the adenosine receptor A_2A_R in the presence of LPS [166]. A_2A_R signaling has been shown to attenuate pro-inflammatory cytokine production [167], suggesting that A_2A_R-expressing M2d cells may be involved in the resolution of the inflammatory response.

##### Mast Cells

Mast cells are commonly found in the dermal layer. Mast cells enter the skin from the bone marrow as progenitors and mature locally in response to environmental cues [168]. While the exact progenitor cell that gives rise to mast cells has been debated, common markers for committed mast cells progenitor cells in the blood are recognized: Lin^-^c-Kit^+^ST2^+^CD34^+^CD16/32^hi^integrinβ7^hi^ [169]. Skin mast cell maturation requires stem cell factor, derived from keratinocytes, while Th2-related cytokines such as IL-3, IL-4, IL-9 and IL-10 induce and promote the proliferation of mast cells [170,171,172]. Mature mast cells are FcεRI^+^c-Kit^+^CD16/32^int^integrinβ7^lo^ [173,174]. Intriguingly, the skin’s microbiome regulates the population of mast cells in the skin. Germ-free mice contain fewer mature mast cells in the dermis, and intradermal injection of these mice with staphylococcal-derived lipoteichoic acid induced the expression of stem cell factor in keratinocytes, ultimately resulting in the rescue of the dermal mast cell population [175]. 

In humans, mast cells are found in all areas of the skin but are most numerous in the arms and the legs [176]. The density of mast cells in the papillary dermis increases with age and they are most often localized in the proximity of PGP9.5+ nerve fibers expressing vasoactive intestinal peptide (VIP), which was shown to suppress mast cell degranulation [177]. This has been associated with the reduction of the amount of extracellular matrix remodeling in the skin observed during the later stages of life. 

Mast cells contain granules containing preformed mediators such as histamine, sulfated proteoglycans, serotonin, and tryptase and/or chymase. In both humans and mice, mast cells resident in the skin express both tryptase and chymase, whereas other tissue-resident mast cells express only tryptase [178]. 

Mast cells are classically known for their involvement in allergic reactions as they produce and release copious amounts of histamine when their Fcε receptors are crosslinked via IgE-antigen complexes [179,180]. They also make large amounts of prostaglandin D2 (PGD2), a lipid-derived inflammatory mediator. The role of PGD2 is nebulous because it has been shown to exert both pro-inflammatory and anti-inflammatory roles. PGD2 is well known to mediate inflammatory responses in asthma [181,182]. However, PGD2 has also been shown to exert anti-inflammatory functions in a murine acute lung injury model as mice that cannot produce PGD2 (H-PDGS^−/−^) exhibited heightened vascular permeability and mRNA levels of pro-inflammatory cytokines in comparison to WT control mice [183].

Mast cells are mass producers of leukotrienes (LTs), LTB4, LTC4, LTD4, and LTE4, with the latter three consisting the cysteinyl leukotrienes (cysLTs). LTs are short-lived lipid inflammatory mediators synthesized via the 5-lipoxygenase (5-LO) pathway. While a multitude of immune cell types express the machinery to produce some of the LTs, mast cells and eosinophils produce the complete battery of LTs. LTB4 has been shown to be effective in attracting neutrophils to sites of inflammation and cell death [63] and is a potent inducer of mast cell degranulation [184]. However, excess LTB4 amounts in the skin can lead to ineffective defense against *Staphylococcus aureus* infections in diabetic mice [185]. CysLTs are classically involved in allergic reactions such as rhinitis [186] and have been shown to activate mast cells in an autocrine manner to induce expression of PGD2, mast cell protease-1 (MCP-1), and histamine in mice and humans afflicted with aspirin-exacerbated respiratory disease [187]. 

A variety of cytokines and growth factors are produced by mast cells either constitutively or in response to a stimulus [188]. Many of these cytokines and growth factors such as TNFα and vascular endothelial growth factor (VEGF) may be preformed and packaged in mature mast cell granules [189,190,191]. Proper formation of mast cell granules is mediated mostly by proteoglycan serglycin [192]. 

Mast cell-derived IL-1β induces production of histamine and IL-8 in human mast cells, suggesting that IL-1β is part of a positive feedback loop for mast cell activation [193,194]. TNFα produced by mast cells is known to drive migration of dermal dendritic cells to draining lymph nodes in a murine model of hapten-induced contact hypersensitivity [195]. Interestingly, mast cell-derived TNFα is also crucial for maintenance of tolerance toward allogeneic skin grafts in mice [196]. Furthermore, recent studies revealed that mast cells are able to form immunological synapses with γδ T lymphocytes when challenged with dengue virus [197] and can acquire MHCII expression via vesicle transfer from dendritic cells after administration with dinitrofluorobenzene [198]. These studies suggest that dermal mast cells can prime adaptive responses during cutaneous infections. 

##### Eosinophils 

Eosinophils are skin-resident cells [199,200], but not much is known about their role in tissue homeostasis. Eosinophilic granules are loaded with potent and toxic proteins: major basic protein (MBP), eosinophil peroxidase (EPO), eosinophil protein X/eosinophil-derived neurotoxin (EPX/EDN), and eosinophil cationic protein (ECP) [61]. Human and murine eosinophilic granules also contain a variety of preformed cytokines and chemokines released in response to appropriate stimuli [201,202,203]. The release of their granular contents is mediated via a process known as piecemeal degranulation [204,205]. This process denotes degranulation in small portions so as to not jeopardize the viability of the cell. Interestingly, cytokines can be chaperoned to secretory vesicles containing their cognate receptors, thus allowing their rapid release from eosinophils [203]. Like mast cells, eosinophils produce all types of LTs and PGD2 [206], with the latter being crucial for eosinophilic infiltration of the skin in hypersensitivity reactions such as atopic dermatitis [207]. In addition, eosinophils generate extracellular DNA traps (EET) containing eosinophil granules [208,209]. These traps are believed to play a role in antibacterial defense. 

Eosinophils are classically known to promote host defense against parasitic infections [210]. Interestingly, eosinophil-lineage deficient mice exhibit no difference in parasitic burden after *Schistosoma mansoni* infection [211]. This seems to be site-specific and eosinophils seem to not play a role in helminth clearance in intestinal sites [212]. However, eosinophils were shown to be necessary in intestinal clearance of adult *Heligmosomoides polygyrus* [213]. Thus, there are still gaps in our understanding of eosinophil-specific responses towards parasitic infections. 

The roles of eosinophils in dermatoses, or skin diseases associated with eosinophilia such as allergic contact dermatitis and urticaria, are well elucidated [61,214,215,216]. In eosinophilic dermatoses, extensive eosinophilic degranulation in the skin results in local tissue damage. 

Eosinophilic pustular folliculitis is an uncommon dermatosis hallmarked by the presence of pruritic follicular papules and pustules associated with folliculotropic infiltration by eosinophils [217]. Skin lesions are most commonly found on the facial area and would typically last for one to two weeks [61]. The recruitment of eosinophils by PGD2 [218] in eosinophilic disorders occurs by two proposed mechanisms. First, by direct binding of PGD2 onto its cognate receptors on eosinophils, thus inducing chemotaxis. Second, by inducing sebocytes to produce eotaxin-3 [219], which then recruits eosinophils. 

Eosinophilic cellulitis, also known as Wells’ Syndrome, is a rare disorder characterized by multiple large, circular, and usually painful or pruritic edematous erythema [61]. While the cause of this disorder is unclear, it has been well documented that the skin of patients with Wells’ Syndrome is infiltrated by eosinophils along with aggregates of complexes comprised of collagen associated with eosinophilic granular contents [220,221].

#### 3.4.2. Lymphoid Immune Cells 

The skin harbors different types of lymphoid cells (Table 1), all of which are important in both steady state and inflammatory responses. Both human and murine skin contain γδ T lymphocytes and αβ T lymphocytes, along with natural killer T cells. γδ T cells are the dominant T cell population in murine skin, while αβ T cells are the dominant T cell population in human skin [222,223]. 

##### αβ T Lymphocytes 

In both mice and humans, αβ T lymphocytes are found in the epidermis and dermis [224], and traffic to the skin from the periphery via cutaneous lymphocyte antigen (CLA) interactions with E-selectin (expressed on endothelial cells), which is upregulated under inflammatory conditions [225,226]. αβ T lymphocytes in the skin are resident memory T cells (T_RM_), which are long-lived and distinct from their circulating counterparts [227]. 

Most T_RM_ in the skin are derived from antigen-specific effector T cells, which previously infiltrated the tissue as a result of an infection. After resolution, these T_RM_ cells seed all areas of the skin but are denser in areas of antecedent infection [228]. Following a skin infection, T_RM_ cells are also found in distal organs such as the lung and gastrointestinal tract [228].

T_RM_ express lower levels of CD28 than effector memory T cells, but can mount robust local recall responses [227] without emigrating from the tissue to do so [229]. Interestingly, T_RM_ also exert sentinel-like functions by promoting recruitment of other memory T cells from the periphery to sites of infection [230,231].

The most studied skin T_RM_ are CD8+ T cells. Skin CD8+ T_RM_ were shown to be effective against HSV infections by inducing an IFNγ-mediated antiviral state in the tissue [230]. Additionally, CD8+ T_RM_ cells were recently shown by the Gebhardt group to exert antitumor functions in a murine transplantable melanoma model [232]. CD8+ T_RM_ cells are usually found in the epidermis and may dislocate dendritic epidermal T cells [233]. All CD8+ T_RM_ express CD69, and a high proportion also express CD103 [233]. CD103 is required for development of CD8+ T_RM_ cells in the skin [233] and mediates adhesion interactions with keratinocytes via an E-cadherin-independent manner [234].

CD4+ T_RM_ cells also make up a significant portion of the skin-resident lymphocyte population and are found in both the epidermis and dermis [233]. Although the function of CD4+ T cells in the skin is not studied to the same extent as CD8+ T_RM_, they are often the dominant αβ T cell subset. During steady state, clusters of antigen-presenting cells with CD4+ memory T cells are found around the hair follicles in both murine and human skin, and cells in these clusters circulate between the skin and periphery [67]. These clusters are formed because keratinocytes in the hair follicles produce IL-7 and IL-15, which are required for homeostatic maintenance of the T cell populations [235]. 

It is also well established that a proportion of skin CD4+ T cells are T regulatory cells (T_regs_). Crosstalk between LCs and T_regs_ has been shown to play a role in dampening immune responses in contact hypersensitivities [236]. T_regs_ dysregulation leads to disorders such as pemphigus vulgaris, alopecia areata, and systemic sclerosis [237,238]. 

Skin-infiltrating and -resident T cells play a role in the pathophysiology of various conditions such as psoriasis, alopecia areata, and vitiligo, in which both CD4+ and CD8+ T cells are involved [239,240,241,242]. In psoriasis, infiltrating T lymphocytes produce IL-17, which is a central part of the disease’s pathophysiology. The hair follicle bulbs and melanocytes are attacked by infiltrating cytotoxic CD8+ T cells in alopecia areata and vitiligo, respectively. CD4+ T_RM_ cells can promote the migration of dDCs from the skin to draining lymph nodes in the context of autoimmune reactions [243].

##### Non-Conventional T Cells 

The mechanisms by which T lymphocytes recognize peptide antigens complexed with the MHC macromolecules are well delineated. However, T cells can also recognize free soluble antigens and non-peptide antigens complexed with non-classical MHC-like molecules, and these T cells are termed non-conventional. Here we will review the types of non-conventional T cells found in the skin:

##### γδ T Lymphocytes: 

Unlike the αβ T lymphocytes, γδ T cells do not undergo the same stringent negative selection process during development and are released from the thymus in waves, with the first wave of γδ T cells seeding the dermis of the skin [244,245,246]. 

Most γδ T cells in murine skin are found in the epidermis within the junctions between keratinocytes, and are known as dendritic epidermal T cells (DETCs). Lymphocytes with DETC-like characteristics have yet to be discovered in humans [222,223]. It was recently shown that DETCs are derived from yolk sac progenitor cells and are self-renewing in the epidermis in a similar manner to LCs [247]. DETCs express a canonical γδ T cell receptor and sample antigens via their dendrites, which can extend from their mid cell body located in the apical epidermis to the border with the stratum corneum, maintaining contact with local keratinocytes [248]. DETCs require keratinocyte-derived IL-7 for survival [249], and IL-15 is also important as DETCs were absent in IL-15^−/−^ mice [250]. On the other hand, DETC-derived IGF-1 and keratinocyte growth factor (KGF) promote keratinocyte proliferation in steady state [251]. It is speculated that DETC-derived homeostatic factors are packaged in granules and transported via the DETCs’ dendrites [252]. 

Once activated, DETCs retract their dendrites, adopt a round morphology, and secrete a range of cytokines and growth factors. IL-17-producing DETCs are essential for inducing the expression of β-defensins in the epidermis, thus playing a critical role in antimicrobial defense of the skin [253]. Interestingly, DETCs were shown to migrate to cutaneous draining lymph nodes in a CCR7-dependent process after sensing stressed keratinocytes in an inducible Notch1 knockout mouse model, thus likely playing a role in antitumor immunity [69]. 

γδ T cells found in the murine dermis express a Vγ4 chain and thus are distinct from their DETC counterparts that express the Vγ5 chain [254]. Additionally, dermal γδ T lymphocytes require IL-7, but not IL-15, for population maintenance [255], and are highly motile in comparison to DETCs that remain in close contact with keratinocytes during tissue homeostasis [256]. The functions of dermal γδ T cells in inflammatory skin conditions are well documented. This population of dermal T cells is inclined to produce IL-17 [256], indicating their significance in cutaneous diseases such as psoriasis [257,258]. 

γδ T cells in the skin can also play a protective role in cutaneous defense, as a previous study showed that γδ T cell-deficient mice exhibited larger lesions and reduced IL-17 production in response to *Staphylococcus aureus* compared to WT control mice [70]. 

γδ T cells including DETCs are not MHC-restricted [259] and can recognize soluble antigens [259,260], antigens derived from damaged or stressed cells [261,262,263], or antigens complexed with non-classical MHC molecules such as CD1b, CD1c, and CD1d [264,265] or MHCI-related chain A/B (MICA/MICB) [266]. A recent study suggests that γδ T cells also recognize Btnl proteins, which are part of the B7 superfamily [267]. 

##### Other CD1-Restricted Cells 

CD1 molecules are essential for presenting lipids antigens to T cells and are categorized under group I (CD1a, CD1b, CD1c) or group II (CD1d and CD1e) [268,269]. In addition to γδ T cells, some αβ T cells and natural killer T cells are CD1 restricted.

Humans contain both group I and group II CD1-restricted T cells, while mice only contain group II CD1-restricted T cells. LCs express CD1a, which was recently shown to regulate Th17-mediated inflammatory responses in the skin [270]. Autoreactive T lymphocytes specific to squalene, wax esters, and triglycerides home to the skin, and these lipids are presented by CD1a [271]. CD1b molecules present a diverse array of mycobacterial lipids such as mycolic acid, glucose monomycolate, and phosphatidyl mannosides [272,273,274,275]. CD1c molecules have been shown to present self-lipid antigens such as methyl-lipophosphotidic acid, which is known to aggregate in leukemic cells [276]. A previous study showed that mice containing autoreactive CD1c-restricted T cells were efficient in eliminating human leukemic cells, suggesting that these autoreactive CD1c-restricted T cells play a role in tumor immunity [276]. Unlike the T_RM_ lymphocytes, CD1-restricted T cells have not been documented to be present in healthy skin, but are readily found in the context of inflammatory conditions. Both CD1b and CD1d have been documented in human cutaneous *Borrelia burgdorferi* infections [277]. Group I CD1-restricted T lymphocytes elicit IgE responses when presented with phospholipid antigens derived from cyprus pollens, suggesting that they contribute to pollen hypersensitivities [278]. CD1a expressed on LCs is central on skin inflammatory conditions such as atopic eczema [270,279] and psoriasis [280]. Furthermore, group I CD1-restricted T cells have been implicated to play a role in controlling the microbiota inhabiting the skin. Group I CD1-restricted T cells have also been well documented in the defense against *Mycobacterium tuberculosis* [281,282].

Group II CD1d-restricted T lymphocytes have been more extensively studied in mice. CD1d is expressed by keratinocytes and dermal dendritic cells [283]. Most group II CD1d-restricted T cells are also known as invariant natural killer T cells (iNKT). iNKT cells are involved in hypersensitivity reactions as they produce IL-4 in response to haptens in the skin, leading to sensitized iNKT cell accumulation [284]. Similar to the group I CD1-restricted T cells, iNKT cells also exhibit antitumor activity [285]. Interestingly, it has also been suggested that iNKT cells play a role in suppressing autoimmune responses in the skin, as a deficiency of CD1d resulted in increased numbers of skin lesions in a MRL-*lpr/lpr* murine model of systemic lupus erythematosus [286]. Furthermore, small non-lipid synthetic molecules can activate T cells via CD1d presentation [287]. CD1e molecules are expressed in the Golgi compartments of immature DCs and are later localized in the lysosomes of mature DCs [288]. CD1e is known to be crucial for the antigen processing of bacterial glycolipids to CD1b-restricted T cells [289]. However, not much is known about the roles of CD1e in cutaneous inflammation.

##### B Lymphocytes 

B cells are rather sparse in the skin in steady state and it is unclear whether they are indeed resident to the skin [290,291,292]. However, the roles of B lymphocytes in skin inflammatory conditions are well documented. In humans, B cells are found in elevated levels in cutaneous diseases such as atopic eczema, cutaneous leishmaniasis, and cutaneous sclerosis [293,294,295]. B cells are found in the reticular dermis over the course of these diseases and are associated with increased levels of IgM, IgE, and IgG. In a similar manner to T lymphocytes, B lymphocytes traffic to the skin tissue via CLA [296]. It has been suggested that the CCL20-CCR6 axis is important in addition to CLA for B cell homing from lymph to the skin [291,297]. A population of B-1-like cells is found in inflamed skin in both humans and mice and migrates from the peritoneum via integrin α4β1 [298]. B cells also play a role in delayed-type hypersensitivity reactions in the skin. Peripheral B-1 cells produce allergen-specific IgM antibodies as soon as day one post-induction with either ovalbumin or keyhole limpet hemocyanin. This leads to the formation of immune complexes between the IgM antibodies and allergens and activation of the complement cascade mechanisms, ultimately resulting in recruitment of T cells to the affected skin site [299,300]. Autoimmune bullous diseases are characterized by the presence of autoantibodies that are reactive to structural proteins of the epidermis [301]. Pemphigus vulgaris is such an example in which desmoglein-3 autoantibodies lead to the formation of blisters in the skin [302]. Intriguingly, a population of regulatory B cells (B_regs_) have been suggested to play suppressive roles in certain cutaneous inflammatory conditions. B_regs_ exert their immunosuppressive functions through the production of large amounts of the regulatory cytokine IL-10 [303]. In an imiquimod-induced murine psoriasis model, mice that lacked most B cells (CD19^−/−^) exhibited more severe symptoms when compared to their WT counterparts. Adoptive transfer of WT B cells to the CD19^−/−^ mice ameliorated psoriasis symptoms, suggesting that a population within these B cells play a therapeutic role in alleviating the disorder [304]. 

#### 3.4.3. Non-Immune Cells 

Pattern recognition receptors (PRR) are expressed by most cells of the skin and have been characterized on keratinocytes, fibroblasts, adipocytes, melanocytes, and endothelial cells [305]. Activation of these receptors results in the production of cytokines and chemokines by non-immune skin cells, thus participating in the local immune response [306,307,308,309]. 

Keratinocyte-derived inflammatory responses have been extensively studied. These cells express almost all intracellular and extracellular PRRs and produce a variety of cytokines, chemokine and AMPs to protect the host against infection [78,79,80]. As mentioned earlier, keratinocytes both in the epidermis and skin elements are in constant interaction with local immune cells and produce factors crucial in homeostasis and in tissue repair [85,235,248,249]. Other studies demonstrated that keratinocytes produce IL-33 in response to hypoosmotic stress [76], and that human and murine keratinocytes produce IL-6 and IL-1β mediated by NFκB signaling in response to UVB irradiation [310]. Evidence has also shown that keratinocytes can produce inflammatory cytokines and chemokines mediated by STAT3 signaling in the presence of IL-25 in a murine psoriasis model, suggesting that keratinocytes play a crucial role in the pathophysiology of the disease [311]. Additionally, keratinocytes have mechanisms in place to control overactivation of inflammatory responses [312].

Fibroblasts immunomodulatory functions have also been well delineated. They express PRRs, synthesize many cytokines, and were shown to produce AMPs [313]. Dermal fibroblasts and keratinocytes produce serum amyloid A in response to PRR signaling [77], which is believed to induce the production of pro-inflammatory cytokines from various immune cells [314]. A recent investigation showed that human fibroblasts cultured in vitro produced massive amounts of TNFα, IL-1β, IL-6, IL-8, and IL-25 when subjected to thermal stress [315]. 

## 4. Innervation of the Skin 

The skin is highly innervated by sensory nerves expressing receptors that can sense pain (nociceptors), itch (pruriceptors), temperature (thermoreceptors), and touch (low-threshold mechanoreceptors; LTMRs) [316]. Nociceptors, pruriceptors, thermoreceptors, and some mechanoreceptors are present as nerve free endings. Other mechanoreceptors are present in the skin as corpuscles. The cell bodies of nerves that innervate the skin are located in the trigeminal and dorsal root ganglia [1]. Nociceptive nerves are in close contact with hair follicles and epithelial cells with their free nerve endings terminating at various levels of the epidermis [317]. Merkel cells are oval-shaped cells involved in mechanosensation (light touch) interspersed in the basal layer of the epidermis and innervated with sensory fibers. Merkel cells are anchored to the epidermis through cytoplasmic protrusions from the Merkel cell to keratinocytes and by desmosomes [316]. Other mechanoreceptors in the skin are Meissner’s and Pacinian corpuscles. Meissner’s corpuscles are localized in the papillary dermis and are sensitive to touch, while Pacinian corpuscles are located in the reticular dermis and are responsive to pressure and vibration [318,319]. Both types of corpuscles are supplied by Aα and Aβ sensory nerve fibers that are situated in the sensory ganglia [319]. Thermoreceptors, critical for sensing thermal differences between the skin and the external environment, are expressed on both heat- and cold-sensitive nerves, with the skin being more densely populated by cold-sensitive nerves [320]. Activation of thermally sensitive nerves to either heat or cold results in vasodilation, vasoconstriction, sweating, or shivering [321]. 

Noxious stimuli are detected by nociceptors, which have been shown to orchestrate local immune responses. The transient receptor potential (TRP) ligated-ion channels are critical detectors of pain-inducing stimuli. Members of the TRP family function as both thermoreceptors and nociceptors. TRPA1 respond to cold stimuli whereas TRPV1 and TRPV2 respond to hot stimuli [322]. Activation of the TRP channels involves an influx of Ca^2+^ and Na^+^ ions through the channel pore, which subsequently leads to neuronal plasma membrane depolarization and opening of the Na_v_/Ca_v_ channels to prime action potential firing of the neuron [323]. 

TRPA1 and TRPV1 also function in pruritus of the skin and control the function of dermal macrophages [324,325]. Activated TRP nociceptors can release calcitonin gene-related peptide (CGRP), which has been shown to modulate immune responses. CGRP has been shown to promote the migration of T cells to inflamed sites by inducing adhesion of T cells to fibronectin, a component of the extracellular matrix [326]. However, CGRP can inhibit macrophage-mediated activation of T cells [327], indicating that CGRP exerts immunoregulatory functions. CGRP also affects immune responses exerted by neutrophils, as Pinho-Ribeiro et al. demonstrated that CGRP inhibits neutrophilic killing of *Streptococcus pyogenes* in a murine model of necrotizing fasciitis [328]. 

TRPV1 has also been implicated in cutaneous disorders. TRPV1 ablation attenuated skin inflammation in a murine model of psoriasis [329]. A recent study showed that histamine increases the sensitivity of TRPV1 in mouse dorsal root ganglia neurons, in vitro [330,331]. There are four known histamine receptors: HR1-HR4, and three of these receptors are expressed on the dorsal root ganglia [332], implicating that activation of these receptors is integral in nociception [333]. Thymic stromal lymphopoietin (TSLP) is a protein produced by epithelial cells and promotes the expansion of lymphocytes and Th2 cells via DC activation [334]. Dendritic cells themselves can also produce copious amounts of TSLP in response to ligation of β-glucans to the receptor Dectin-1 [335]. Interestingly, TSLP has also been shown to activate skin nociceptive sensory nerves and is highly associated with pruritus [336]. 

Remarkably, immune cells in the skin can regulate the innervation of the tissue. A previous study demonstrated that depletion of langerin+ cells in langerin-DTR mice via diphtheria toxin treatment exhibited a significant reduction in the numbers of PGP9.5- and CGRP-expressing nerves, and a decrease in the levels of nerve growth factor (NGF) and glial-cell-line-derived neurotrophic growth factor (GDNF) [337]. Furthermore, TNF-producing mast cells induce growth of nerve fibers in mice when subjected to oxazolone-induced contact hypersensitivity [338].

## 5. Skin Microbiome 

Commensal microorganisms including bacteria, fungi, and viruses are found in the epidermis, dermis, and dermal appendages, and constitute an additional layer of defense for the host. Their communities are fairly stable over time but are site- and individual-specific [339]. The microbiome colonizes the skin early after birth in an immune-regulated process [340]. By occupying accessible areas, the microbiome regulates colonization of the tissue by pathogenic microorganisms [341], promotes homeostatic immunity [342] and modulates gene expression [343]. 

The major commensal microbes inhabiting the skin are *Staphylococcus epidermidis* (*S. epidermidis*) and *Propionibacterium acnes* (*P. acnes*) [341]. Commensals also protect the host by competing for habitable space, thus preventing colonization of the skin by pathogenic microbes. Colonization of the skin by pathogenic strains is usually associated with low presence of commensal stains [344]. Some of these commensal strains can secrete their own antimicrobial agents, such as bacteriocins, which inhibit the growth of pathogenic bacterial strains [345]. The Gallo group found that coagulase-negative *S. epidermidis* on human skin produces its own AMPs that are protective against the pathogenic *Staphylococus aureus* (*S. aureus*) colonization [346]. Commensals can also indirectly inhibit the growth of pathogenic strains. *P. acnes* in wounded mice fermented glycerol into short-chain fatty acids, that inhibited the colonization of the wound by the pathogenic methicillin-resistant *S. aureus* (MRSA) likely through a process that resulted in lowering the intracellular pH of MRSA [347]. Furthermore, commensal *S. aureus* protects against pathogenic MRSA by inducing antibody production against α-hemolysin [348]. 

Commensal bacteria can manipulate the host’s immune response. Cogen et al. showed that *S. epidermidis* secretes delta toxin, which can induce the formation of neutrophil extracellular traps and promote AMP activity against group A *Streptococcus* [349]. Lipoteichoic acid derived from commensal *S. epidermidis* recruited mast cells to the skin via TLR2 signaling when challenged with vaccinia virus [350]. Remarkably, the same lipoteichoic acid produced from *S. epidermidis* can ameliorate tissue pathology in mice exposed to poly I:C via TLR2-mediated inhibition of TLR3, suggesting that commensals can reduce tissue damage after an injury by suppressing inflammation [351]. Linehan et al. recently found that *S. epidermidis* antigens are presented to CD8+ T lymphocytes, and that these lymphocytes are capable of producing IL-17 (Tc17) or IFNγ (Tc1) [352]. The *S. epidermidis*-responsive Tc17 and Tc1 populations were also shown to be restricted to the non-classical antigen presenting molecule MHCIb. Commensal bacteria occupying hair follicles may be responsible for the clustering of memory T lymphocytes to the vicinity. It is thought that these resident bacteria induce the production of chemokines and cytokines by hair follicle keratinocytes, thus promoting the recruitment of immune cells [235,353]. 

Surprisingly, commensals may play a role in promoting antitumor immunity. Nakatsuji et al. demonstrated that the *S. epidermidis* produce 6-N-hydroxyaminopurine (6-HAP), which suppresses melanoma growth in C57BL/6 mice, and protects against UV-induced neoplasia in SKH-1 hairless mice [354]. The same study also found that 6-HAP is produced by *S. epidermidis* inhabiting human skin. 

The fungi kingdom in the skin is not very diverse and the most commonly found species is *Malassezia* spp. [355,356]. *Malassezia* cannot synthesize its own lipids and therefore is mostly found on sebaceous skin, where it can convert the skin’s endogenous lipids into free fatty acids, suggesting that *Malassezia* contributes to cutaneous defense [357]. However, *Malassezia* may also be involved in promoting skin pathologies. A recent study showed that most patients with atopic dermatitis exhibit some degree of sensitization to *Malassezia* antigens [358]. Furthermore, *Malassezia* has been implicated to play a role in causing dandruff and seborrheic dermatitis via endogenous lipase activity [359]. 

Viruses also reside in healthy skin. The viral microbiome (virome) is not well delineated and although not as diverse as the bacterial kingdom, it exhibits more diversity than that of fungi [360]. In humans, one of the common viruses found in the skin is the Merkel cell polyomavirus (MCV), which is found in about 60% of adults [361]. Two other polyomaviruses have been found on human skin: human polyomaviruses-6 and 7 (HPyV6 and HPvV7) [362]. While not much is known about the functions these commensal viruses exert on healthy skin, individuals with compromised immune responses can be afflicted with tumors such as Merkel cell carcinoma that are associated with the polyomaviruses [363].

## 6. Skin Immune Responses in Wound Healing

**Figure 1:**
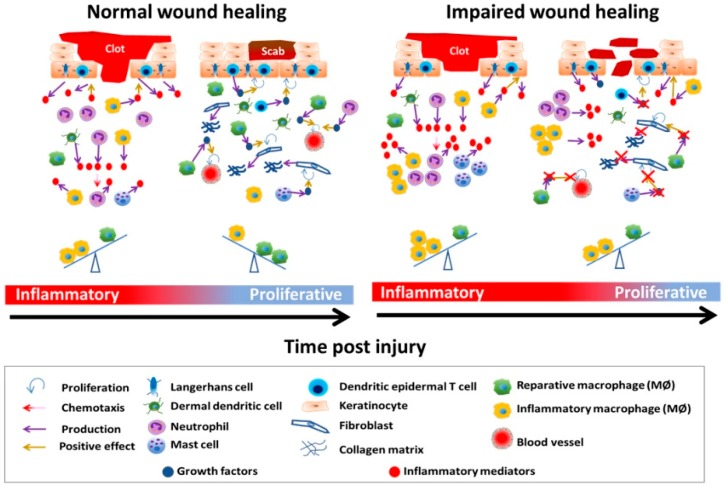
Schematic of normal and impaired wound healing. The inflammatory phase is hallmarked by infiltration of the wound by activated immune cells. Chemotaxis of immune cells to the injury site is facilitated by the presence of inflammatory mediators, which can be produced by skin-resident cells such as keratinocytes, dermal dendritic cells, Langerhans cells, and macrophages. The proliferative phase is characterized by expansion of keratinocytes and endothelial cells to restore the barrier function of the skin and vasculature of the tissue, respectively. Fibroblasts are major producers of collagen fibers in the wound bed and a source of de novo synthesized extracellular matrix. Efficient wound healing is characterized by the timely transition from the inflammatory to the proliferative phase. For this to occur, several events have to happen in concert: neutrophil numbers in the wound decrease, macrophages shift in phenotype from inflammatory to reparative, collagen deposition, and revascularization, thus facilitating wound closure. Chronic wounds are characterized by high levels of inflammation, and decreased production of growth factors, decreased proliferation of endothelial cells, and lack of re-epithelialization. For simplicity, the remodeling phase, certain immune cell types such as tissue-resident T lymphocytes, and nerve fibers are omitted. Wound complications such as bacterial infections and scarring are also omitted.

The wound healing process consists of four tightly orchestrated and largely overlapping phases: hemostasis, inflammation, proliferation, and remodeling (Figure 1). 

In **hemostasis**, the skin tissue isolates the injured area from the environment and prevents further bleeding by forming a clot. In the case of an insult, tissue factor (TF), normally located in the subendothelial spaces of the skin, is exposed to blood via compromised vasculature. This initiates the coagulation cascade, during which platelets adhere to components of extracellular matrix constituents, secrete their granular contents, and aggregate [364,365]. Platelets release granular contents that consist of coagulation factors IV, V, and VIII, platelet-derived growth factor (PDGF), calcium, serotonin, histamine, and epinephrine. Collectively, these granular contents all play a role in further platelet activation and aggregation, which seals off the injured area. At the same time, zymogen forms of plasminogen and thrombin are converted into functional enzymes [366]. The wound clot consists of fibrin and serves as a scaffold for keratinocytes to begin re-epithelialization and for immune cells to infiltrate the wound area [7,81,366,367]. 

The **inflammatory** phase is characterized by infiltration of the wound by immune cells, such as neutrophils, monocytes, and lymphocytes. The milieu of the wound during this phase consists of high levels of pro-inflammatory mediators, which serve to recruit other immune cells from the periphery. The purpose of the inflammatory phase is for the host to ward off any pathogens that have entered the wound site and prevent infections. In addition, phagocytes clear out necrotic debris. 

The **proliferative** phase is marked by expansion of skin-resident cells including keratinocytes, fibroblasts, and endothelial cells [368]. During this phase, keratinocytes expand and migrate to restore the barrier function of the epidermis. Granulation tissue, a matrix of immune cells, fibroblasts, and de novo generated blood vessels, begins to form and replaces the fibrin clot, which forms the substrate for migrating keratinocytes to adhere to. Blood that was matrixed with the fibrin clot forms a scab, which serves as an outer shield for the underlying migrating keratinocytes [81]. Fibroblasts differentiate into myofibroblasts, which are responsible for providing contractile forces in wound closure [369]. The Horsley group showed that IL-22 promotes fibroblast activity during wound healing by inducing collagen deposition in murine wounds [370]. Remarkably, it was recently discovered by Guerrero-Juarez et al. through single cell mRNA-seq analyses that fibroblast populations in the wound are heterogenous and consist of up to 12 different subsets [55], with some of these subpopulations being localized in different areas of the dermis. Both fibroblasts and myofibroblast begin producing a collagen network consisting of immature type III fibers that act as the foundation of the new extracellular matrix [7,81]. 

In the **remodeling** phase, the injured tissue attempts to restore its original architecture. Many immune cells, endothelial cells, and myofibroblasts undergo apoptosis or are removed from the wound, leaving mostly the newly developed extracellular matrix and collagen fibers. Macrophages that remain in the wound at this phase produce and secrete matrix metalloproteases (MMPs) that are involved in the remodeling of the extracellular matrix by removing any excess collagen [81]. The removal of excess collagen by MMPs also serves to reduce any scar tissue that forms after injury. Furthermore, the newly synthesized immature collagen type III fibers from the proliferative phase begin maturing into the terminal collagen type I conformation [371]. During this phase, LCs begin to repopulate the neo-epidermis [51,372]. Once initiated, the remodeling phase can continue for at least one year post-injury [81,366].

The immune response is central during the entirety of the wound healing process (Table 1). Shortly after the injury, neutrophils, the first cells to arrive at the wound, are crucial for defense again pathogenic microbial colonization of the wound. During inflammatory responses, neutrophils release neutrophil extracellular traps (NETs) in a process called NETosis, which assist in the immobilization of pathogens. The magnitude of NETosis determines whether the process results in death or viability of neutrophils [373]. NETs are complexes containing histones, granular enzymes and peptides including neutrophil elastase, defensins, and cathelicidins [373,374]. Recently, it was discovered that neutrophils produce and secrete coagulation factor XII, which can be used to induce NETosis in an autocrine manner, and can recruit other neutrophils from the periphery [62]. Neutrophils can further amplify the inflammatory immune response by secreting potent chemoattractants such as IL-8 and LTB4, which recruit other neutrophils to the injury site [63,375]. In addition to their inflammatory roles, neutrophils secrete factors that promote healing, such as laminin 5 β-3, which allows keratinocytes to adhere to the dermal layer of the wound bed [64]. Intriguingly, a small subset of neutrophils has been identified to be pro-angiogenic and is responsive to VEGF-A in both humans and mice [65,66]. These pro-angiogenic neutrophils may be categorized under a unique phenotype, coined as “N2”, in a similar manner to the M1 and M2 macrophages, by Fridlender et al. during an investigation of tumor-associated neutrophils [376].

Interestingly, catecholamines have been documented to play a role in the neutrophilic response during wound healing. The Isseroff group showed that wounded mice that were treated with epinephrine exhibited significant increases in the neutrophil population in the wound site, suggesting that catecholamines mediate trafficking of neutrophils to injury sites [377]. Blockade of catecholamine signaling decreased the local neutrophil population [377] and promoted wound healing [378,379]. 

Monocytes from the periphery infiltrate the wounded tissue shortly after neutrophils. Depending on the environmental milieu, monocytes can give rise to M1 or M2 macrophages, or dendritic cells once they arrive at the site of inflammation [150]. Recently, the Gurtner and Plikus groups demonstrated that wound-infiltrating F4/80+ monocytes can give rise to new fibroblasts, suggesting that monocytes are a source of de novo fibroblasts in injured skin [55,380]. Macrophage subsets exert multiple functions such as clearance of invading pathogens, secretion of growth factors that can stimulate revascularization and tissue repair, and expression of metalloproteases that are involved in restoration of the tissue architecture [368,381,382]. M2 macrophages produce VEGF, which promotes angiogenesis [163,383], promote fibroblast proliferation and secrete TGFβ and PDGFβ, which collectively lead to accelerated tissue repair [381]. In addition, macrophages in the wound bed secrete cytokines and growth factors that drive the proliferation and activation of a population of myofibroblasts that express similar markers to those of adipocyte progenitor cells [56], which are known to be critical for wound healing [384]. Interestingly, depletion of macrophages has been associated with impaired healing [157,385,386], and this reparative effect is likely exerted later in the wound healing process, concomitant with their switch towards M2 function [387]. It is not quite clear what exactly causes the switch of macrophages towards an M2-like (pro-reparative) phenotype. Previous studies demonstrated that phagocytosis of apoptotic cells, such as neutrophils [388,389], by macrophages induces signals that initiate the resolution of inflammation, a process known as katabasis, and the return of the tissue to homeostatic functions [388,390,391]. In support of this, a recent study in a murine myocardial infarction model showed that mice depleted of neutrophils exhibited a reduction in the MerTK+ M2c macrophages, which are involved in clearing of apoptotic cells and debris [392]. However, studies have shown that neutrophils do not always undergo apoptosis at the wound site and have been documented to exit the wound area and migrate via the lungs to the bone marrow, where they are eliminated [393]. 

LCs begin to repopulate the neo-epidermis during the remodeling phase of healing [51,372], and a study in humans showed that patients who exhibit efficient healing contained higher densities of LCs in the skin [394]. The source of de novo LCs in healing tissue can be either the local pre-existing LC population in unwounded tissue or from blood-circulating monocytes [395]. In mice, stressed keratinocytes upregulate ligands for lymphocyte activation receptor natural killer group 2D (NKG2D), such as Rae-1, which leads to the migration of LCs from the epidermis [51,396]. Interestingly, mice that do not express NKG2D ligands (Klrk1^−/−^) exhibit a delay in wound healing, suggesting that crosstalk between keratinocytes and LCs is important for efficient wound healing [397]. 

Expansion of dendritic cells in the skin enhances healing by promoting re-epithelialization, angiogenesis, granulation tissue formation, growth factor production [53], and promotes skin wound healing via the MiR-21/PTEN signaling axis [398]. pDCs infiltrate the wound bed during the inflammatory phase and secrete IFNα/β [143]. It was shown that antibody-mediated depletion of pDCs in murine wounds results in a decrease of the inflammatory response, but also a reduction in wound closure [143].

Lymphocytes also play a crucial role in wound healing. In mice, DETCs have been shown to be associated with wound healing as they can affect keratinocyte proliferation and epidermal thickening by secreting KGF [72]. Human Vδ1-expressing γδ T cells are activated after injury and secrete IGF-1, which promotes the healing process [399]. A previous study by the DiPietro group showed that CD4+ and CD8+ T lymphocytes can influence the wound milieu by regulating the mRNA expression of various cytokines and growth factors, but does not significantly affect wound closure [400]. Regulatory T cells have been implicated in wound inflammation as a study demonstrated that depletion of T_regs_ using a FoxP3-DTR murine wound model resulted in increased inflammatory population of Ly6C+ monocytes in the wound, leading to impaired healing [401]. T_regs_ also play a role in the maturation of newly formed vasculature as depletion of T_regs_ in mice resulted in a decrease in the numbers of mature endothelial cells associated with myofibroblasts after excision injury [68]. 

iNKT cells have recently been documented to play a role in wound healing as iNKT-deficient mice (Jα18KO) exhibited an increase in neutrophilic inflammation of the wound, resulting in delayed healing when compared to WT mice [73].

MicroRNAs (miRNAs) are RNA sequences of approximately 22‒23 nucleotides and have been studied extensively in the past. The roles of miRNAs in cutaneous wound healing are now becoming more understood and appreciated. The most studied miRNA in wound healing is miR-21, which is highly expressed in keratinocytes and fibroblasts. It has been shown that inhibition of miR-21 results in impaired healing in mice [402]; however, blockade of this miRNA in humans has the opposite effect [403]. Other miRNAs of interest in wound healing are miR-29 [404] and miR-210 [405], and further research is needed to fully elucidate the roles of these miRNAs in wound healing. 

The skin maintains a net negative charge relative to the tissue laying underneath it. In the case of an injury, ion leakage occurs, creating a voltage gradient throughout the wound site [406]. The application of a voltage gradient in the wound activates signaling molecules that are critical for wound healing such as integrins, epidermal growth factor receptors, and phosphoinositide 3 kinases [407,408]. The migration of cells by endogenous bioelectrical cues, known as galvanotaxis or electrotaxis, can occur at the site of injury [409]. Many cells are responsive to electrical fields, including epithelial cells, fibroblasts, lymphocytes, macrophages, endothelial cells, and neuronal cells [406], although these cells migrate in different manners. For instance, stromal fibroblasts migrate toward the anode whereas epithelial cells migrate toward the cathode [410,411]. A recent study by Nakajima et al. showed that galvanotaxis/electrotaxis of human corneal epithelial cells is largely influenced by the purinergic receptors P2X and P2Y [412]. Electrical fields may also play a role in the migration of stem cells, which are crucial for efficient wound healing. A previous in vitro study showed that murine adipose-derived stem cells are responsive to electrical currents and their migratory speed is increased proportionally to the strength of the applied electric field [413], suggesting the potential use of electrotherapy in promoting migration of stem cells to injury sites. Interestingly, a recent in vivo study showed that wounded mice that were treated with a bioelectric plaster exhibited accelerated healing, which was, however, associated with a thickened epidermis in comparison to mice that received no treatment [414]. Previous clinical studies have assessed the efficacy of various electrotherapies (direct current, pulsed current) in accelerating the healing of lower extremity wounds [415]. One such study reported that venous leg wounds treated with a low direct current for six weeks resulted in enhanced healing [416]. A more recent study showed that treatment of pressure ulcers with pulsed current electrotherapy in supplementation with standard wound care significantly increased wound healing in elderly patients [417]. 

Ageing skin exhibits changes in its intrinsic properties and reduced ability to restore itself after an injury. Some of these changes include decreases in barrier function, dermal thickness and fibroblast numbers, tissue microvasculature density, and insulation ability [418]. Keyes et al. showed that epidermal cells proliferate at a slower rate in aged mice after injury, and that aged keratinocytes were slower at re-epithelialization in scratch wound assays [419]. 

### 6.1. Impaired Healing 

Disruptions in any of the phases of wound healing result in impaired healing. A prolonged inflammatory phase may result in chronic wounds and inefficient wound healing [420] (Figure 1). Perturbed proliferative and remodeling phases may lead to irregular wound closure, fibrosis, and scarring [81]. Moreover, some chronic wounds express elevated levels of MMPs, which can impair the formation and compromise the structural integrity of the fibrin clot [421,422,423]. Impaired healing results in increased hospitalization time, higher costs, and lower quality of life. Venous stasis ulcers, arterial stasis ulcers, pressure ulcers and diabetic wounds are the most common non-healing chronic wounds. Venous stasis ulcers are associated with tissue inflammation and iron overload [424], which is suggested to impair M1 macrophage transition to M2 [425]. Additionally, the cytokine environment may impair fibroblast function [424,425]. Arterial leg ulcers occur as a result of reduced arterial blood flow and subsequent tissue perfusion. Also, pressure ulcers are observed in patients with low mobility due to tissue necrosis and capillary damage [426,427,428]. In this section, we will focus on diabetic wounds in which immune deregulations have been extensively studied. In addition, we will discuss burns, an inflammatory type of wound usually associated with deregulated healing.

#### 6.1.1. Diabetic Wounds 

Diabetic patients suffer by complications such as neuropathies, vascular diseases due to poor blood circulation, and perturbed immune responses leading to opportunistic infections [429]. The most common wound in diabetic patients are those that affect the feet. Diabetic foot wounds may not be detected due to concomitant neuropathies, which reduce the patients’ nociception. As a result of neglect, compounded by complications of diabetes, the initial wound in the foot becomes a slow-healing ulcer [430,431]. In extreme cases, significantly delayed healing may lead to amputation of the affected extremity. The skin of diabetic patients exhibits disruptions in mechanisms that are central to maintaining homeostasis and structural integrity. Diabetic skin is associated with reduced VEGF expression [432] and recruitment of smooth muscle cells [433], implicating that the vasculature of the tissue is perturbed. Studies in diabetic mouse models showed that hyperglycemia disrupts the barrier function of the skin, reduces epidermal proliferation, and decreases homing of endothelial progenitor cells from the bone marrow to the skin [434,435]. TF levels in diabetic skin are decreased in comparison to normal skin, implying that the coagulation cascade that occurs immediately after wounding is dampened [436]. Topical application of TF on wounds of diabetic mice seems to improve healing, although it does not improve healing to the same efficiency as that of non-diabetic wounds [436]. Proliferation and migration of keratinocytes in diabetic skin are dampened in high-glucose environments [437,438], indicating that wound closure is decelerated in diabetic skin. 

In both diabetic mice and patients, wounds contain neutrophils that undergo excessive NETosis, usually leading to collateral tissue damage [374,439]. Interestingly, macrophages in diabetic wounds are dysfunctional, which results in inefficient neutrophil clearance [440], chronic inflammation [441], and impaired growth factor production [442], and consequently inefficient healing. Importantly, transfer of bone-marrow-derived M2 polarized macrophages were shown to improve healing in mice that were hyperglycemic due to hyperplastic beta cells [443]. Inflammatory lipid mediators such as leukotrienes are also found in diabetic wounds and it is speculated that they prolong inflammation, thus impairing wound healing [185,444]. Higher numbers of mast cells have been observed in the skin of diabetic patients in comparison to non-diabetics [445], and prevention of mast cell degranulation by disodium cromoglycate treatment accelerated healing in diabetic mice when compared to untreated diabetic mice [445].

The current standard of care for slow-healing wounds is to debride the injury site. This is done in an attempt to reveal viable tissue and to provide space for the tissue to proliferate and expand [446]. Sometimes a moist dressing is used on the injury site in an effort to support the inflammatory phase of wound healing and re-epithelialization of the wound [447]. In addition, acellular and cell-loaded matrices are used in many cases of chronic wounds with varying degrees of success [448]. 

A variety of mechanisms that accelerate healing in diabetic wounds have been identified over the years. VEGF accelerates healing by recruiting endothelial progenitor cells to the skin of mice that are hyperglycemic, due to a genetic deletion in the leptin receptor (Lepr^db/db^) [449]. Intriguingly, diabetic mice that received folic acid orally showed increased levels of hydroxyproline, a major component of collagen, and enhanced re-epithelialization, suggesting that folic acid increases collagen turnover and wound closure [450]. Hydrogen sulfide has also been shown to exert therapeutic effects on wound healing in Lep^ob^ mice, by attenuating inflammation at the injury site [451]. Furthermore, the potential of mesenchymal stem cells (MSCs) derived from bone marrow, adipose tissue, or amniotic fluid in treatment of chronic wounds has been widely explored [452]. The therapeutic potential of these cells is associated with their ability to induce anti-inflammatory responses and secrete proangiogenic agents and growth factors [452]. In addition, MSCs-derived exosomes carrying miRNA, or growth factors are currently being explored as potential treatments [453]. These recent advances in diabetic and chronic wound healing have the potential of bringing novel therapeutic treatments to the clinic.

#### 6.1.2. Burns 

Burn injuries are categorized by degrees. A first-degree burn injures the epidermal layer of the skin, followed by a second-degree, which affects the dermis, and lastly a third-degree injury that goes as deep as the subcutaneous tissue. Burn injuries are characterized by an intense inflammatory phase and edema. Blistering is also commonly found in burn patients with second-degree injuries. However, blistering is absent in murine and porcine skin, which are commonly used as burn injury animal models.

Severely burned patients usually present a variety of systemic complications, such as depressed or aggravated immune responses, electrolyte imbalance, sepsis and multiple organ dysfunction syndrome [454,455,456,457], and inflammation-associated psychological effects [458]. In this section, we will focus on responses that take place locally at the burn wound site and how these affect healing. 

In addition to initial damage occurring at the time of the injury, tissue destruction continues both in size and in depth as a result of extensive secondary necrosis due to impaired vascularization, free radical formation, and inflammatory mediators [459,460]. The burn site is inundated with inflammatory mediators and immune cells that exacerbate local tissue damage and further delay wound closure [461,462,463]. Inhibition of several cytokines has been shown to promote healing in burns [464,465]. 

Immune cells that infiltrate a burn wound are phenotypically the same as those that infiltrate a non-burn wound. However, it is not clear whether the functionality of the immune cells that enter the burn wound is the same as those in non-burn wounds. Interestingly, although neutrophils have been documented to populate burn wounds in high numbers and for prolonged periods of time, studies have shown that their functions are impaired [466,467,468,469]. Activation and migration of neutrophils is essential for pathogen clearance at the wound site. Activated neutrophils may also migrate in distal organs where they exert damaging effects [470,471,472]. Although depletion of neutrophils leads to increased infection rates, treatment of rats with resolvin D2 within the first week after burn injury restored neutrophil migration [473]. In addition, prostaglandin E2 (PGE2) levels at the wound site dictate a pro-repair neutrophil phenotype [474]. Interestingly, the presence of CD11c+Ly6G+ neutrophil/DC hybrid cells has been documented in burn wounds [53] with yet undefined roles. The formation of CD11c+Ly6G+ cells has been observed in other inflammatory conditions in mice, and this subset may be suggestive of neutrophil plasticity [475]. The mechanism by which neutrophils or neutrophil subsets influence burn wound healing requires further exploration. 

Mast cells are key players in burn injuries and studies have shown they may orchestrate both the inflammatory and the proliferative phases. They are among the first responders in burn injury and release histamine and cytokines to promote the inflammatory phase [476]. Deletion of mast cells reduces tissue damage in a second-degree scald burn injury model in mice [477]. Interestingly, their numbers are shown to increase after burn injury and reach their peak at later stages, suggesting they may also be involved in the proliferative phase [478]. It is suggested that mast cells promote tissue repair and remodeling during the later stages of burn wound healing [478,479]. However, uncontrolled mast cell activity has been associated with fibrosis and scar formation [57,480].

Monocytes enter the burn site a few days after neutrophils and locally mature to macrophages, initially exhibiting a pro-inflammatory phenotype. In animal models, a CCR2+ monocyte/macrophage population is associated with the inflammatory phase, while CX3CR1+ monocyte/macrophage population is associated with the proliferative phase [481]. Other than secretion of growth factors [162,163], macrophages also interact with local cells such as fibroblasts to promote healing [482]. Granzyme K expressed by macrophages is elevated in burn patients, and genetic deletion of granzyme K (GzmK^−/−^) in mice results in improved healing, suggesting that granzyme K activity in macrophages plays a major role in burn wound healing [483].

The role of dendritic cells at the wound site is not well delineated. Depletion of all CD11c+ cells during the early stages post-burn in CD11c-DTR mice resulted in delayed wound healing accompanied by decreased local cell proliferation and vascularization [53]. However, whether there are specific roles assigned to dermal dendritic cells subsets in burn injury is still not known.

A burn injury usually results in the formation of an eschar. Blood flow in the tissue underneath an eschar is restricted resulting in ischemia and further tissue damage [461]. However, hypoxic environments promote wound closure by adipocyte-derived progenitor cells in an in vitro scratch wound assay [484]. Burn wounds are characterized by decreased levels of hypoxia-inducible factor 1α (HIF1α) [485]. Interestingly, HIF1α haploinsufficiency results in impaired wound healing in mice after burn injury, during the later stages of healing and was associated with decreased angiogenesis compared to HIF1α-sufficient mice [486], corroborating the notion that HIF1α is crucial during the proliferative stage of burn wound healing. Expression of HIF1α on Tie2+ cells (endothelial and bone-marrow-derived myeloid cells) mediates the recruitment of angiogenic cells to the burn site as Tie2Cre-HIF1α^fl/fl^ mice exhibited significantly reduced numbers of de novo generated CD31+ vessels in the burned wound bed [487]. Additionally, hypoxic monocyte-derived LCs support Th1 responses, ultimately affect healing progression [488]. 

T cell responses are dysregulated in burn injuries, likely contributing to impaired healing. The levels of IL-17 and IL-22, cytokines that support Th17 responses, are significantly elevated in burned mice when compared to sham controls [489]. The activity of skin-resident γδ T lymphocytes is also aberrant. While known for their roles in promoting wound healing [72], γδ T cells have been shown to upregulate expression of the cytokines IFNγ, IL-17, and IL-10 as soon as three days post-burn in mice, suggesting that γδ T cells perpetuate Th1, Th17, but also regulatory T cell responses in the burn site [490]. Remarkably, γδ T cells mediate the infiltration of wounds by αβ T cells [491,492], but αβ T cells in burn wounds exhibit an inactivated phenotype as shown by the decreased expression of CD69 [492]. 

Deregulated proliferative and remodeling phases are commonly seen in burns resulting in scarring and/or keloid formation (discussed later in this review). However, it has yet to be elucidated whether this is an independent event or the result of a deregulated inflammatory phase. 

Currently, the standard of care for severely burned patients is to undergo autologous split thickness skin or allograft grafting [461,493]. However, these procedures can lead to complications such as pain at the grafted site, blood loss, impaired donor skin integration followed by bacterial infection and inflammation [494,495], and in the case of allograft, rejection. In addition to auto- and allografts, skin substitutes are also in development [496]. This is an exciting area of research that has produced promising results. However, the lack of skin elements and innervation in these substitutes can cause the patient a multitude of complications such as lack of sensation, perspiration and motion flexibility. Cellular or acellular matrices are also employed, albeit with inconsistent results. Thus, the need for developing new strategies that promote endogenous healing and tissue reconstruction is dire. 

Similar strategies employed for improving healing in chronic wounds have been tested in burn wounds. Burn wounds treated with umbilical cord-derived MSCs showed improved healing compared to control wounds [497]. Furthermore, bone-marrow-derived MSCs (BM-MSCs) were shown to migrate to the site of the burn injury via a CXCR4‒CXCL12 signaling axis and improve healing [498]. Many studies have addressed the role of MSCs in burn wound healing, and some of the pro-healing functions seem to be increased by ex vivo manipulation such as hypoxic culture and viral transduction for VEGF, before transplantation [499,500]. Phototherapy is also investigated as a method to promote burn wound healing. A recent study showed that rats with second-degree burns exhibited faster healing that was associated with increased levels of autophagy as shown by upregulated LC-3 I and LC-3 II expression followed by suppression of the inflammasome when the wounds were subjected to far-infrared phototherapy [501]. 

Immunological responses in burn injuries differ with age. A clinical study revealed a trend in which elderly patients expressed higher levels of pro-inflammatory cytokines but contained fewer macrophage counts in the wound bed when compared to the younger group [502]. Although the authors did not evaluate the wound re-epithelialization, they did show a trend in which older patients were associated with a higher burn severity score index when compared to the younger patients.

The sensation of pain in burn injuries may also play a role in controlling local inflammation. It was recently shown that the TRPV1 nociceptor expressed on sensory nerves in the skin releases CGRP in response to burn injury, which then regulates the expression of pro-inflammatory cytokines. This effect can be attenuated when fibulin-5, an extracellular matrix protein found in endothelial and smooth muscle cells, is overexpressed [503]. 

## 7. Wound Complications 

Impaired wound healing may lead to severe complications such as local or systemic infections, neuronal damage, and scarring or keloid formation. We will discuss these complications below.

### 7.1. Infections 

The milieu of non-healing wounds is characterized by deregulated immune responses and as such, it facilitates colonization of the wounded tissue by pathogenic bacteria [504,505]. Biofilms, commonly formed in non-healing wounds, are single- or multi-strain communities of microbes embedded in an extracellular matrix composed of carbohydrates, extracellular DNA, and proteins. This complex structure confers the biofilm’s resistance to antibiotics and immune responses by preventing entry of small molecules and immune cells [505,506,507]. Diabetic wounds are associated with a variety of antibiotic-resistant bacterial strains [508,509] such as *S. aureus*, *Escherichia coli* (*E. coli*), *Klebsiella*, and pathogenic forms of *S. epidermidis* [504]. Burn patients are also commonly afflicted with bacterial infections, with the most common strains being *Klebsiella pneumoniae*, *Acinetobacter baumanii*, *Pseudomonas aeruginosa*, and *S. aureus* [510,511,512]. Many of these strains can form biofilms, and if these infections persist, may lead to bacteremia and ultimately to sepsis [513,514]. Previous studies have postulated that the dysregulated immune response in sepsis could be due to simultaneous activation of pro-inflammatory and anti-inflammatory mechanisms [515,516]. Aftereffects of sepsis may last for a lifetime if the host survives the initial event. Mice that survived sepsis demonstrated severe splenomegaly and an elevated population of Ly6C+ inflammatory monocytes [517]. Those who cannot clear wound infections risk tissue necrosis, potential amputation of the affected extremity, and death from septic shock.

### 7.2. Nerve Damage and Chronic Pain 

Wounds that are extensive in skin depth usually result in nerve damage. After initial damage, distal axons degenerate in a process known as Wallerian degeneration. This is followed by formation of a growth cone, which is usually located at the tip of the proximal bud of the injured nerve. Filipodia sprout from the growth cone for the regenerating nerve to sample the microenvironment. The path of the growth cone is affected by the presence of scar tissue in the wound bed, and the growth cone releases proteases to clear its path [518]. In steady state, sensory nerves require neurotropic factors for survival and maintenance. These factors are also required for the efficient repair of damaged nerves. Interestingly, a source of brain-derived neurotropic factor in mice with facial nerve transection was the cervical lymph nodes, suggesting that immune cells can secrete neurotrophic factors [519]. Schwann cells in the periphery are important cells in nerve regeneration as they also provide neurotrophic factors needed to promote regeneration [520]. Remarkably, macrophages have been indicated to play a role in Schwann cell function after nerve injury [521]. Patients with nerve damage suffer partial or complete loss of sensory or motor functions in the affected area, numbness, and pain. A transected nerve can be debilitating for patients who have suffered lacerations as the muscle surrounding the injured nerve may undergo atrophy if the injured area remains denervated for extended periods of time, during the healing process [522]. Burn injuries, especially third-degree burns, usually exhibit nerve damage resulting in complete loss of sensation at the affected site. During nerve repair, the immune response shifts toward an anti-inflammatory phenotype. Mice with a transected sciatic nerve exhibited an increase in the mRNA encoding genes associated with anti-inflammatory/pro-reparative macrophages [523]. 

Similar to wound healing, the age of the patient may be a factor in the efficient nerve regeneration. Aged mice with a peripheral nerve crush injury healed slower, exhibited a higher number of Iba1+ macrophages near the affected area, and sustained higher expression of pro-inflammatory markers when compared to the younger control mice, indicating that aging impairs nerve damage repair [524]. 

Pain is normally associated with inflammation. However, extensive inflammation and tissue damage may result in persistent pain, which is associated with alterations in the dorsal root ganglia and the spinal dorsal horn. In chronic pain, the central nervous system upregulates COX-2 via p38 MAPK pathway in response to pain due to substance P injection, suggesting that eicosanoids contribute to chronic pain [525]. TNFα has been shown to reduce the mechanical threshold of C-nociceptors, which can lead to constant activity and hypersensitivity [526]. Furthermore, TNFα levels are elevated in the spinal cord after peripheral nerve injury, corroborating the suggestion that inflammatory cytokines are fundamental to chronic pain [527]. In the spinal cord, the voltage-gated sodium channels Nav1.3, Nav1.7, and Nav1.8 have been suspected to be active in chronic pain [528]. Patients who suffer from nerve damage and chronic pain experience lower quality of life due to restrictions in movement, decreased activity in daily life functions, and increased irritability.

### 7.3. Hypertrophic Scarring and Keloids 

Hypertrophic scarring is the result of overproduction of collagen in the wound bed by fibroblasts. Scar tissue is characterized by lack of skin elements such as hair follicles and sebaceous glands, and consequently, stem cells that typically populate these elements [529]. Hypertrophic scarring is common in burns and cutaneous injuries that affect the dermal layer and may adversely affect the patient’s quality of life [530]. 

Hypertrophic scarring can be disfiguring and as such is associated with emotional and mental effects, and in extreme cases, with self-harm or suicide [531,532,533,534]. Scarring can also restrict movement when occurring in areas such as the hands, knee, elbow, neck, and face [495]. The etiology of hypertrophic scars is still not well understood. However, it has been shown that it is associated with reduced collagenase activity, increased presence of myofibroblasts, and high levels of TGFβ and PDGF [535,536,537]. Previous studies have shown that fibroblasts in the wound bed exhibit increased expression of TGFβ and the TGFβ1 receptor, indicating that these cells may be hypersensitive to TGFβ signaling and thus produce copious amounts of collagen observed in scar tissue [538]. Accordingly, human fibroblasts in which the TGFβ receptor was knocked down had reduced production of extracellular matrix components [539]. 

Intriguingly, macrophages that do not express β-catenin lack the ability to adhere to fibroblasts and fail to induce TGFβ production, suggesting these macrophages induce scar formation by providing TGFβ needed to activate fibroblasts [540]. 

The potential use of stem cells in reducing scarring has been demonstrated as introduction of BM-MSCs reduced fibrosis in a murine bleomycin-induced fibrosis model [541]. Mast cells have been implicated in fibrosis and scarring [58,59,60], making them a potential target for therapies. Polyurethane scaffolds are also in consideration in the reduction of hypertrophic scars as implantation of a scaffold in debrided murine burn injuries reduced the rigidity of the formed scar tissue [542].

In humans, keloids are also a common complication characterized as hypertrophic dermal elements. The collagen content of keloids consists of type III and type I collagen fibers while hypertrophic scars consist of mostly type III collagen [543,544]. Additionally, hypertrophic scars appear shortly after injury and may reduce in size over time, while keloid development may lag, and their size can extend beyond the boundaries of the original wound. Both keloids and hypertrophic scars require TGFβ [545]. Crosstalk between keratinocytes and fibroblasts has been implicated in a previous in vitro study in which keloid-derived fibroblasts induced the expansion of co-cultured keratinocytes [546]. 

Current treatments for both hypertrophic scars and keloids involve the use of occlusive dressings to provide hydration to the affected site, steroid treatment supplemented with compression therapy to reduce collagen cohesiveness, surgical excision, cryosurgery and radiation therapy [545]. Additionally, emerging therapies include immunomodulation, botulinum toxin A, hydrogel scaffolds, and skin tension offloading devices [545]. 

Another type of treatment that was recently developed and has shown promise, both in promoting wound healing and reducing scarring, is mechanotherapy. A few of these therapies are silicone gel sheets, paper tape, and the “Embrace advanced scar therapy” [547]. Silicone gel sheets are used to reduce tensile stresses in the wound area and maintain hydration to the epidermis, which results in mediation of the wound cytokine microenvironment [548,549]. Treatment with silicone gel sheets has also been documented to reduce the expression of TGFβ in fibroblasts [550]. Use of paper tape reduces wound tension. Paper tape reduced hypertrophic scarring in women who had undergone cesarean section, compared to women who did not receive treatment [551]. Embrace scar therapy involves a silicone sheet-based polymer dressing device that is used to apply compressive forces to wound sites. In a clinical trial conducted by the Gurtner group, patients who were treated with the Embrace device after undergoing abdominoplasty showed a significant improvement in scar appearance when compared to patients in the control treatment group [552]. 

To reduce the scarring or keloid formation in patients, investigators have studied the mechanisms of scarless healing observed in fetal skin. Previous studies have elucidated a variety of factors that distinguish fetal from adult wound healing. Fetal wound sites contain fewer inflammatory immune cells than adult wounds, thus reducing the amount of collateral tissue damage that occurs during the inflammatory phase of healing [553]. Fetal skin exhibits an increased ratio of type III to type I collagen in comparison to adult skin [81,553]. Hyaluronic acid is elevated in the fetus [554] and extracellular matrix components such as fibronectin are more rapidly deposited in the fetal wound bed [555], suggesting that the microenvironment and resident fibroblasts in fetal wounds are shifted toward a regenerative phenotype. As expected, the number of stem cells in the fetal skin is higher than that in adult skin [556], indicating that regenerative wound healing occurs at a higher frequency in fetal skin than adult skin. Keratinocytes derived from fetuses that were in the mid-gestation period of development were shown to induce the expression of MMPs and collagen production from mature fibroblasts when co-cultured in vitro [557], revealing a potential role of the crosstalk between keratinocytes and fibroblasts in reducing scar formation. 

## 8. Conclusions 

The skin is the largest organ and is in constant contact with the environment. To protect the host from infection, it has developed a variety of defensive strategies. In addition to physical, microbiological, and chemical barriers, the skin contains resident immune cells that serve sentinel functions and contribute to tissue homeostasis. In the event of an insult, these cells act in harmony to locally initiate inflammatory responses, and if needed, to prime adaptive immunity. 

Despite intensive research in the wound healing field, we have yet to fully understand the mechanisms that promote this process. Impaired wound healing gives rise to complications such as persistent infections, neuronal damage, and pain. Additionally, dysregulated inflammatory responses contribute to uncontrolled skin cells proliferation leading to fibrosis. Recent research has delved into the mechanisms of impaired wound healing and uncovered the potential for exploiting cellular and molecular targets for the development of novel therapeutics in hopes of accelerating the healing of chronic wounds. In addition to the approaches discussed in this review, a new approach is the modulation of wound microbiome, discussed elsewhere [558,559,560]. In view of the challenges associated with management of chronic and inflammatory wounds, it is clear that new strategies are needed. 

Understanding the cellular and molecular interactions between immune cells and skin-resident cells is necessary to delineate the dynamics of wound repair.

## Figures and Tables

**Table 1:** Summary of the skin’s immune cells. The location of each immune cell type in the skin tissue and their functions during homeostasis, inflammation, and wound healing are described. N/D: not defined.

Immune Cell Type	Location in Skin	Functions During Homeostasis	Inflammatory Functions	Functions during Wound Healing
**Langerhans cells and dDCs**	Langerhans cells: Epidermis dDCs: Papillary dermis	Sampling of environmental antigens Migrate to lymph nodes to induce tolerance to self-antigens [51] Control of commensal-specific T cells in the skin [52]	Migration to lymph nodes to induce adaptive immune responses to specific antigens Induce T cell responses Produce pro-inflammatory cytokines and chemokines to recruit peripheral immune cells	Migrate to draining lymph nodes to prime adaptive responses Promote angiogenesis, re-epithelialization, formation of granulation tissue, and growth factor production [53]
**Macrophages/monocyte-derived macrophages**	Papillary and reticular dermis	Hair follicle regeneration/maintenance [54] Phagocytosis of cellular debris	Produce cytokines, chemokines to recruit peripheral immune cells Inflammatory macrophages produce inflammatory cytokines (IL-1β, TNFα, IL-6) Phagocytosis of pathogenic agents and necrotic debris	Reparative macrophages produce growth factors (VEGF, TGFβ) and regulatory cytokines (IL-10) Give rise to de novo fibroblasts and induces their proliferation [55] Expression of MMPs during tissue remodeling Activation of myofibroblasts [56]
**Mast cells**	Papillary and reticular dermis	N/D	Produce inflammatory mediators involved in allergic responses and asthma, and recruitment of immune cells Produce inflammatory cytokines and secrete histamine during contact-hypersensitivities	Induce collagen production (fibrosis) from fibroblasts [57,58,59,60]
**Eosinophils**	Reticular dermis	N/D	Defense against parasites Degranulation; release of EPO, MBP, EPX/EDN, and ECP Infiltrates the skin tissue during eosinophilic dermatoses [61]	N/D
**Neutrophils**	Reticular dermis	N/D (not abundant in healthy skin)	Phagocytosis of invading pathogens Release of NETs (NETosis) to immobilize pathogens Production and secretion of coagulation factor XII to induce NETosis [62] Release chemoattractants to recruit other neutrophils to inflamed sites [63]	Secretion of laminin 5 β-3 to induce keratinocyte adhesion [64] Responsive to VEGF and induce angiogenesis [65,66]
**αβ T lymphocytes**	CD8+: Epidermis CD4+: Epidermis and papillary dermis	Sentinels that can recruit other lymphocytes to the skin Found to localize around hair follicles, perhaps to control commensal populations in the proximity [67]	Induces antiviral state in the skin through IFNγ mechanism T effectors produce cognate cytokines (i.e., IFNγ, IL-4, IL-17 T_regs_ suppress inflammatory monocytes and other autoreactive immune cells	Resolution of wound inflammation through T_reg_-mediated control of inflammatory monocytes [68]
**γδ T lymphocytes (DETCs and dermal)**	DETCs: Epidermis Other γδ T cells: Papillary dermis	Secretion of KGF and IGF-1 to maintain keratinocyte populations Migrates to draining lymph nodes after sensing stressed keratinocytes; antitumor immunity [69]	Produce IL-17 to induce β-defensin expression from keratinocytes Protective against cutaneous *S. aureus* infection [70] Involved in disorders such as psoriasis [71]	Secretion of KGFs and IGF-1 to induce expansion of the epidermis [72]
**Non-γδ CD1-restricted lymphocytes**	Papillary and reticular Dermis	N/D	Involved in defense against extracellular pathogens Suppress autoreactive cell activity in systemic lupus erythematosus	iNKT cells may be involved in wound healing by controlling inflammatory neutrophil populations [73]
**B lymphocytes**	Reticular Dermis	N/D	Involved in delayed-type hypersensitivity reactions Involved in cutaneous autoimmune diseases via production of autoantibodies specific to components of the skin [74,75] IL-10-producing B_regs_ suppress autoreactive lymphocyte activation	N/D
**Non-immune cells (i.e., keratinocytes and fibroblasts)**	Epidermis and reticular dermis	Contribute to barrier function of the skin Produce collagen network to provide structural integrity	Produce inflammatory cytokines during disease (i.e., psoriasis), osmotic stress, or irradiation [76,77] Produce AMPs in response to bacterial detection [78,79,80]	Migrate to close wound (re-epithelialization) and restore barrier function [7,81] Synthesize collagen fibers and other extracellular matrix components in the wound bed
